# Resveratrol displays anti-inflammatory properties in an ex vivo model of immune mediated inflammatory arthritis

**DOI:** 10.1186/s41927-018-0036-5

**Published:** 2018-10-10

**Authors:** S. Lomholt, A. Mellemkjaer, M. B. Iversen, S. B. Pedersen, T. W. Kragstrup

**Affiliations:** 1 Department of Biomedicine, Aarhus University, Aarhus, Denmark; 2 Department of Endocrinology and Internal Medicine, Aarhus University Hospital, Aarhus, Denmark; 3 Department of Rheumatology, Aarhus University Hospital, Aarhus, Denmark; 4 Department of Internal Medicine, Randers Regional Hospital, Randers, Denmark

**Keywords:** Resveratrol, Anti-inflammatory agents, Methotrexate, Rheumatoid arthritis, Spondyloarthritis, Low disease activity, MCP-1, Monocyte chemoattractant protein 1

## Abstract

**Background:**

Resveratrol is a natural polyphenol found in berries, roots and wine that is well known to have anti-inflammatory and anti-oxidative properties. The anti-inflammatory effect has been reported for both immune cells and connective tissues, but only few studies have investigated effects on immune mediated inflammatory arthritis. None of which have studied this effect when combining resveratrol with methotrexate or adalimumab, two major drugs in the treatment of immune mediated inflammatory arthritis.

We therefore aimed to investigate the anti-inflammatory effect of resveratrol alone and in combination with methotrexate or adalimumab in ex vivo models of immune mediated inflammatory arthritis. We furthermore aimed to describe any variations in this effect based on disease activity and cellular composition of the synovial fluid infiltrate.

**Methods:**

Synovial fluid mononuclear cells from patients with rheumatoid arthritis (*n* = 7) and spondyloarthritis (*n* = 7) were cultured for either 48 h or 21 days. In both models, synovial fluid mononuclear cells were treated with resveratrol alone or in combination with methotrexate or adalimumab. Monocyte chemoattractant protein 1, matrix metalloproteinase 3 and tartrate resistant acidic phosphatase were measured to quantify inflammation, enzymatic degradation and osteoclast differentiation, respectively.

**Results:**

Resveratrol reduced monocyte chemoattractant protein 1 production by synovial fluid mononuclear cells significantly (*p* = 0.005) compared to untreated controls. The effect of resveratrol was greatest in cultures from patients with low disease activity, i.e. DAS28CRP ≤ 3.2 (*p* = 0.022), and in cultures dominated by lymphocytes (*p* = 0.03). Further, the combination of methotrexate and resveratrol significantly reduced monocyte chemoattractant protein 1 levels compared with methotrexate alone in cultures from patients with low disease activity (*p* = 0.016), and in cultures with high lymphocyte count (*p* = 0.011). Resveratrol did not significantly affect matrix metalloproteinase 3 and tartrate resistant acidic phosphatase production.

**Conclusion:**

Resveratrol has anti-inflammatory properties in our ex vivo model of immune mediated inflammatory arthritis. Results show an additive effect of resveratrol, when combined with methotrexate in samples dominated by lymphocytes and samples from patients with low disease activity. This suggests further investigations in vitro and whether this effect may also be present in a clinical setting.

**Electronic supplementary material:**

The online version of this article (10.1186/s41927-018-0036-5) contains supplementary material, which is available to authorized users.

## Background

The natural polyphenol resveratrol (RSV) is found in certain berries, grapes and plants [1] were it exerts anti-fungal and anti-oxidative effects [2]. In humans, RSV is also shown to possess anti-inflammatory and anti-oxidative properties [1]. The anti-inflammatory effect has been shown in both immune cells [3–6] and connective tissues [7–10]. Importantly, several studies have deemed RSV non-toxic and generally well tolerated [11]. This makes RSV an interesting candidate add-on treatment of immune mediated inflammatory arthritis.

Rheumatoid arthritis (RA) and spondyloarthritis (SpA) are chronic immune mediated inflammatory diseases [12, 13]. Treatment involve synthetic and biologic disease modifying anti-rheumatic drugs such as methotrexate (MTX) and the tumor necrosis factor alpha (TNF-α) inhibitor adalimumab. This treatment is often effective but also comes with some side effects including nausea and increased risk of infection and patients can experience pain and discomfort even when the disease activity is low.

The pathology of both RA and SpA is characterized by synovial inflammation and changes in bone and cartilage [14]. Furthermore, numerous cytokines and enzymes play important roles in the pro-inflammatory signaling, cellular recruitment and tissue changes in both RA and SpA [14, 15]. Examples are monocyte chemoattractant protein 1 (MCP-1) which is associated to recruitment of monocytes in RA [16] and correlated with the swollen joint count in RA patients [17] and matrix metalloproteinase 3 (MMP3) which is involved in degradation of the extracellular matrix [18] and have been identified as an independent predictor for radiographic progression in SpA and RA [19, 20]. Structural changes in the bone/cartilage junction involves inflammation triggered osteoclastogenesis [14] and synovial fluid mononuclear cells (SFMC) from RA and SpA patients are shown to have the potential to differentiate into functional osteoclasts in vitro [21].

The anti-inflammatory effect of RSV is mediated through several different signaling pathways [22]. One of which, the intracellular nuclear factor kappa-B pathway [23] is inhibited via an increase in sirtuin 1 expression and activity [4, 5]. Clinical trials have shown an anti-inflammatory effect in cardiovascular disease [24, 25], metabolic health [26], and in response to cigarette induced low-grade inflammation [27]. The noticeable amount of conflicting literature present is, however, suggested to be due to heterogeneity of designs, populations, dosages etc. [28]. No clinical trials have to the authors knowledge investigated effects of RSV in patients with RA or SpA.

The effect of RSV in arthritis is not fully understood. In vitro studies show that RSV reduces the production of TNF-α and interleukin 1 beta (IL-1β) in monocytes/macrophages from RA patients [4] and inhibit T-cell activation [5]. RSV also inhibits proliferation of fibroblast-like synoviocytes [29] and reduce expression of MMP3 [8] and receptor activator of nuclear factor-κB ligand [9]. In human chondrocytes, RSV exerts an anti-inflammatory and anti-apoptotic effect by inhibiting production of IL-1β and reactive oxygen species [30]. Animal studies have shown a significant anti-inflammatory and pannus inhibiting effect of RSV in rats with adjuvant and antigen induced arthritis [31, 32].

## Methods

This study aims to contribute to the understanding of RSV effects through our ex vivo models of immune mediated inflammatory arthritis, especially regarding disease activity and cellular distribution in synovial fluid. The goal is to describe RSV’s intrinsic effect and compare it to the effects of methotrexate (MTX) and adalimumab. Furthermore, the study seeks to investigate possible additive effects of RSV when added to MTX and adalimumab treatment.

### Study subjects

Subjects were included from patients who contacted the outpatient clinic with at least one swollen joint. Synovial fluid and peripheral venous blood were extracted from each subject, and 14 were randomly selected for cell culturing in this study. The group consisted of seven patients with RA and seven patients with peripheral SpA. Anthropometric data, smoking history and previous treatment were registered for most of the patients. The Disease Activity Score 28 with c-reactive protein (DAS28CRP) was accepted by the authors as the best available measure for disease activity since all patients presented with peripheral disease. For each in vitro culture, the percentage of monocytes and lymphocytes was measured. Synovial fluid cell count before separation of mononuclear cells was not performed.

### Isolation of cells

Peripheral blood and synovial fluid samples were collected in ethylenediamine tetra acetic acid tubes. SFMCs were isolated with Ficoll-Paque (GE Healthcare, Little Chalfont, UK) density-gradient centrifugation and cryopreserved at − 135 °C.

Osteoclasts were grown as previously described [21, 33]. Briefly, thawed SFMCs were cultured in Dulbecco’s modified Eagle’s medium, 10% fetal calf serum, penicillin, streptomycin and glutamine. Cultures were seeded with a cell density of 10^6^ cells/ml and kept in a humidified incubator at 37 °C and 5% CO_2_. Medium was replaced every 3–4 days during the total of 21 days.

### Cell cultures

The two SFMC in vitro models used in this study were cultured for 48 h and 21 days, respectively. The 48 h SFMC culture was run as a model of inflammation and the 21 days SFMC culture as a model of inflammatory osteoclastogenesis. Supernatants were carefully harvested after the designated incubation period and centrifugation at 1200 rpm for 5 min. Cells were cultured under seven different conditions. Medium alone and medium with dimethyl sulfoxide acted as negative controls. Treatment conditions were RSV dissolved in dimethyl sulfoxide (Cayman Chemical, USA, 25 μM), MTX (Ebetrex, Sandoz, 0.5 μg/ml), MTX + RSV, adalimumab (Humira, Abbvie, 5 μg/ml) and adalimumab + RSV.

### MCP-1, MMP3 and TRAP assays

Concentrations of monocyte chemoattractant protein-1 (MCP-1) (Biolegend) and MMP3 (R&D Systems) were measured by commercial enzyme-linked immunosorbent assay (ELISA) to quantify inflammatory changes [16] and the potential for cartilage degeneration [18], respectively. Osteoclast differentiation was assessed with a tartrate resistant acid phosphatase (TRAP) enzymatic assay (B-bridge International).

### Data and statistics

Figures and statistical analysis were done with GraphPad Prism 7.04 for PC (GraphPad software). Results on MCP-1, MMP3 and TRAP were, for most of the analysis, transformed to ratios to achieve normality and reduce inter-donor variation. Paired t-test and Wilcoxon matched pairs test were used when appropriate to compare effects of different culture conditions. Unpaired t-test and Mann Whitney u test were used when analyzing grouped results within a culture condition. Correlations analysis was performed with Pearson’s R or Spearman’s Rho depending on the distribution of data. A two-sided *p*-value of less or equal to 0.05 were considered significant.

MMP3 concentrations were below detection level in five 48 h SFMC cultures and excluded from the analysis. TRAP activity was undetectable in six untreated 21 days SFMC cultures, suggesting an insufficient osteoclast differentiation. Therefore, only eight 21 days SFMC cultures were included in the analysis.

As part of the analysis, patients were divided into subgroups based on DAS28CRP (disease activity), and the cell culture monocyte/lymphocyte composition. DAS28CRP group cutoff was 3.2, which distinguishes low and moderate disease activity. Monocyte and lymphocyte grouping was based on the median and distinguished in the text as “low” and “high”. Monocyte/lymphocyte subgroup analysis was only carried out with subgroups of five or more cultures. DAS28CRP was missing for four 48 h SFMC cultures. Monocyte count was missing for one 48 h SFMC culture.

## Results

### Viability and differentiation of cells

**Fig. 1:**
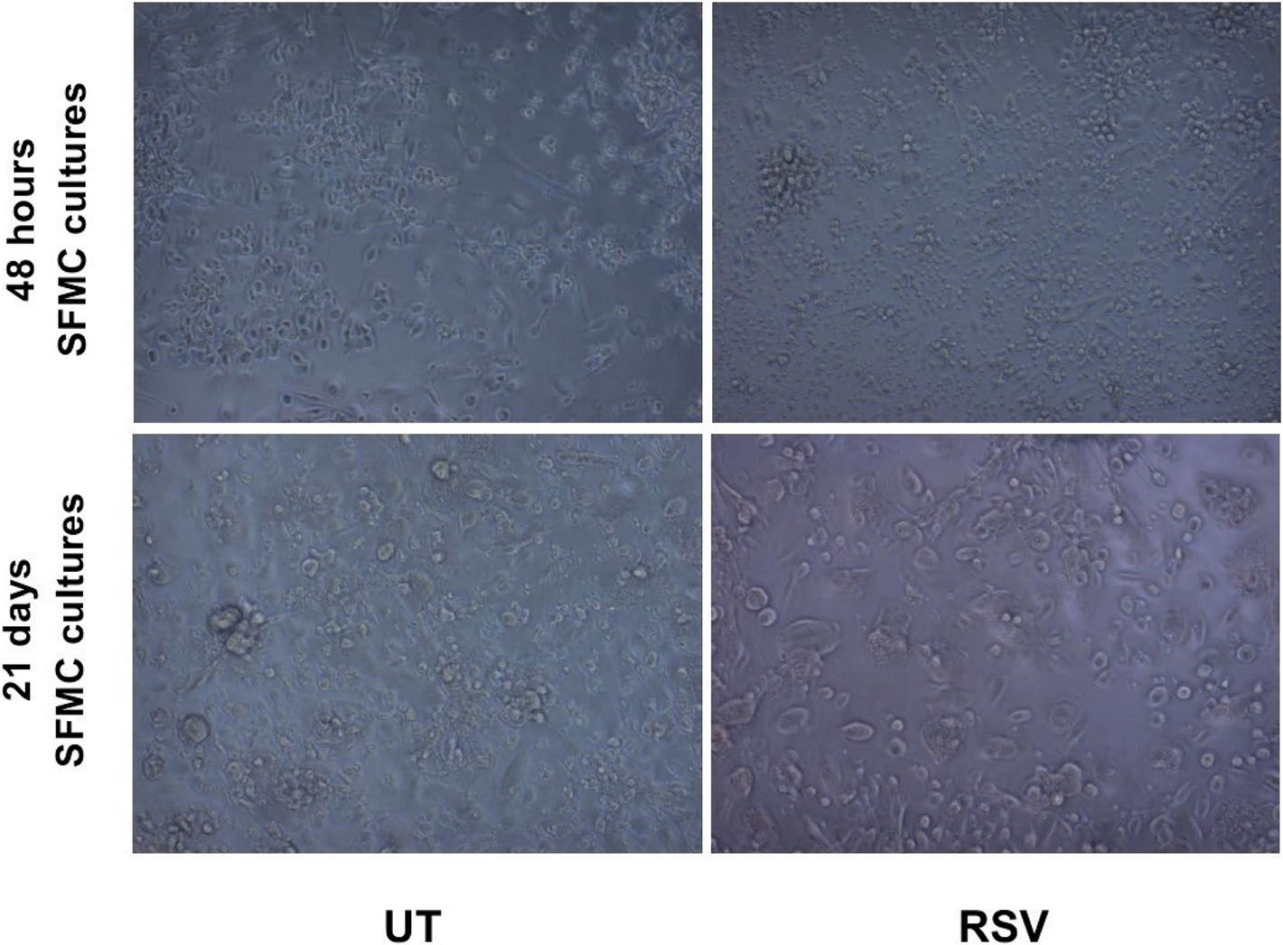
Cell culture viability assessed in microscope (× 10) of 48 h- and 21 days SFMC cultures after incubation period. No immediate cell loss after RSV treatment (25 μM) compared to untreated (UT) cultures. 21 days SFMC cultures showed forming of multinucleated cells

48 h- and 21 days SFMC cultures were assessed under a microscope for viability after the incubation period. Fig. 1 shows images of untreated and RSV treated cultures. Viable cells were present in similar densities for both. No visible cell toxicity was detected in medium with dimethyl sulfoxide compared to medium alone. Furthermore, 21 days SFMC cultures showed presence of multinucleated cells, as shown previously [21]. Enzyme analysis on untreated 21 days SFMC cultures showed TRAP activity in 8 cultures. An additional file shows similar cell viability in 48 h and 21 days SFMC cultures that was untreated or treated with RSV, MTX and adalimumab; Additional file 1.

### Resveratrol significantly decreased MCP-1 concentration in 48 h SFMC cultures without differences comparing response in RA and SpA patients

**Fig. 2:**
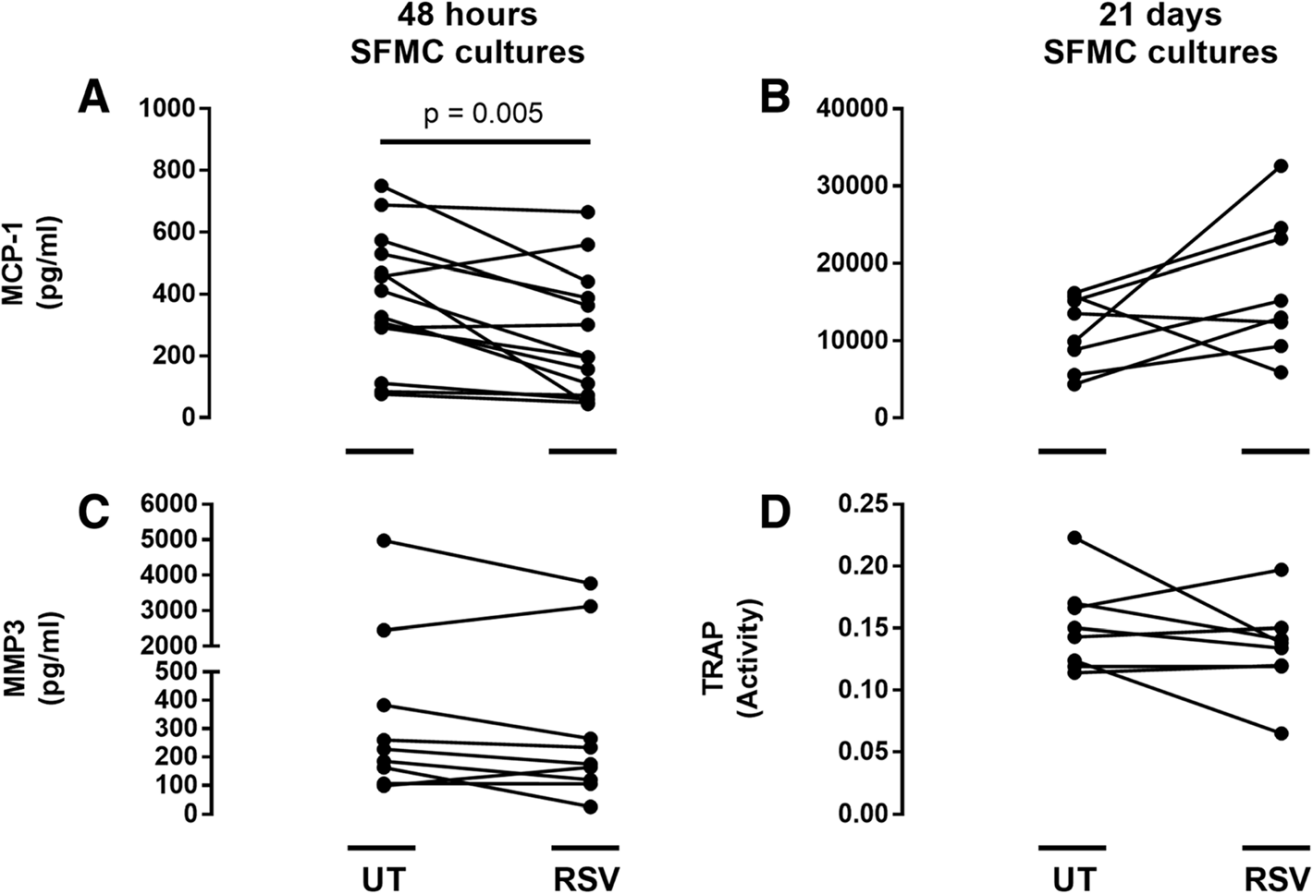
Concentrations of MCP-1 (**a**, *n* = 14 and **b**, *n* = 8), MMP3 (**c**, *n* = 9) and TRAP activity (**d**, *n* = 8) in untreated (UT) and resveratrol (RSV) treated cultures measured by ELISA and enzyme activity assay in their respective supernatants. UT is presented as a negative control to its in-patient corresponding RSV treated culture, * *p* < 0.05. RSV concentration was 25 μM

**Fig. 3:**
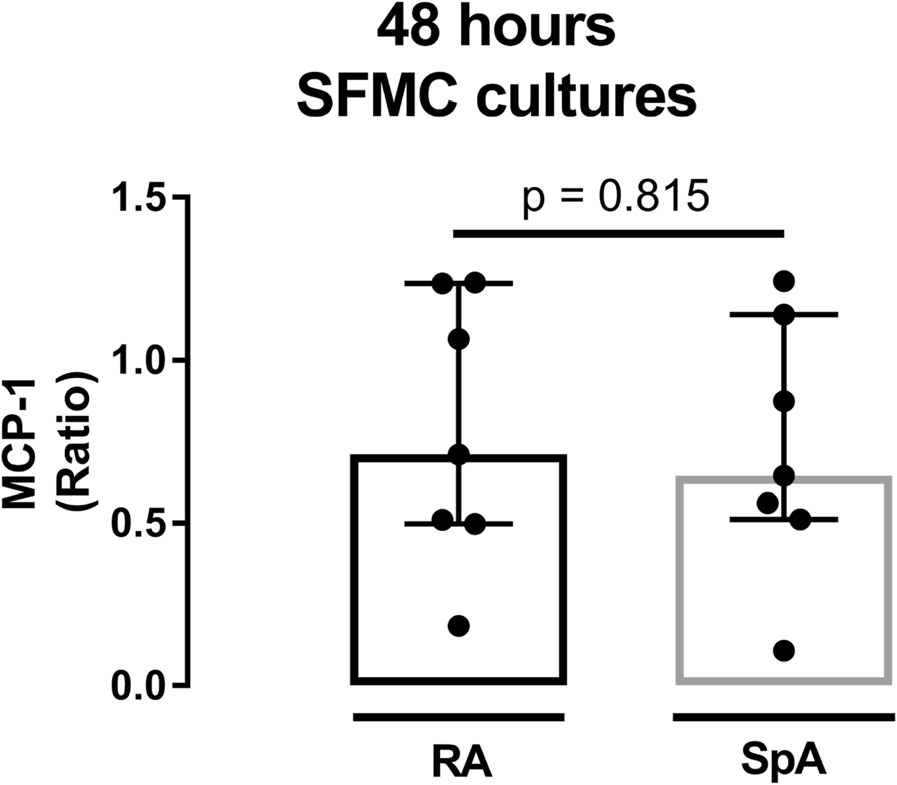
Ratio of MCP-1 in resveratrol (RSV) treated cultures (*n* = 14) divided in groups of patients with rheumatoid arthritis (RA) and spondyloarthritis (SpA). MCP-1 was measured by ELISA. RSV concentration was 25 μM

The measured concentrations/activity of MCP-1, MMP3 and TRAP in culture supernatants are presented in Fig. 2. RSV significantly decreased the concentration of MCP-1 in 48 h SFMC cultures (Fig. 2a, *p* = 0.005), and showed no significant difference when comparing MCP-1 ratios in samples from patients with RA and SpA (Fig. 3, *p* = 0.815). RSV did not change MMP3 concentration in 48 h SFMC cultures or MCP-1 concentration and TRAP activity in 21 days SFMC cultures (Fig. 2b-d).

### Resveratrol’s effect may be influenced by the level of disease activity and the cellular subset in synovial fluid

**Fig. 4:**
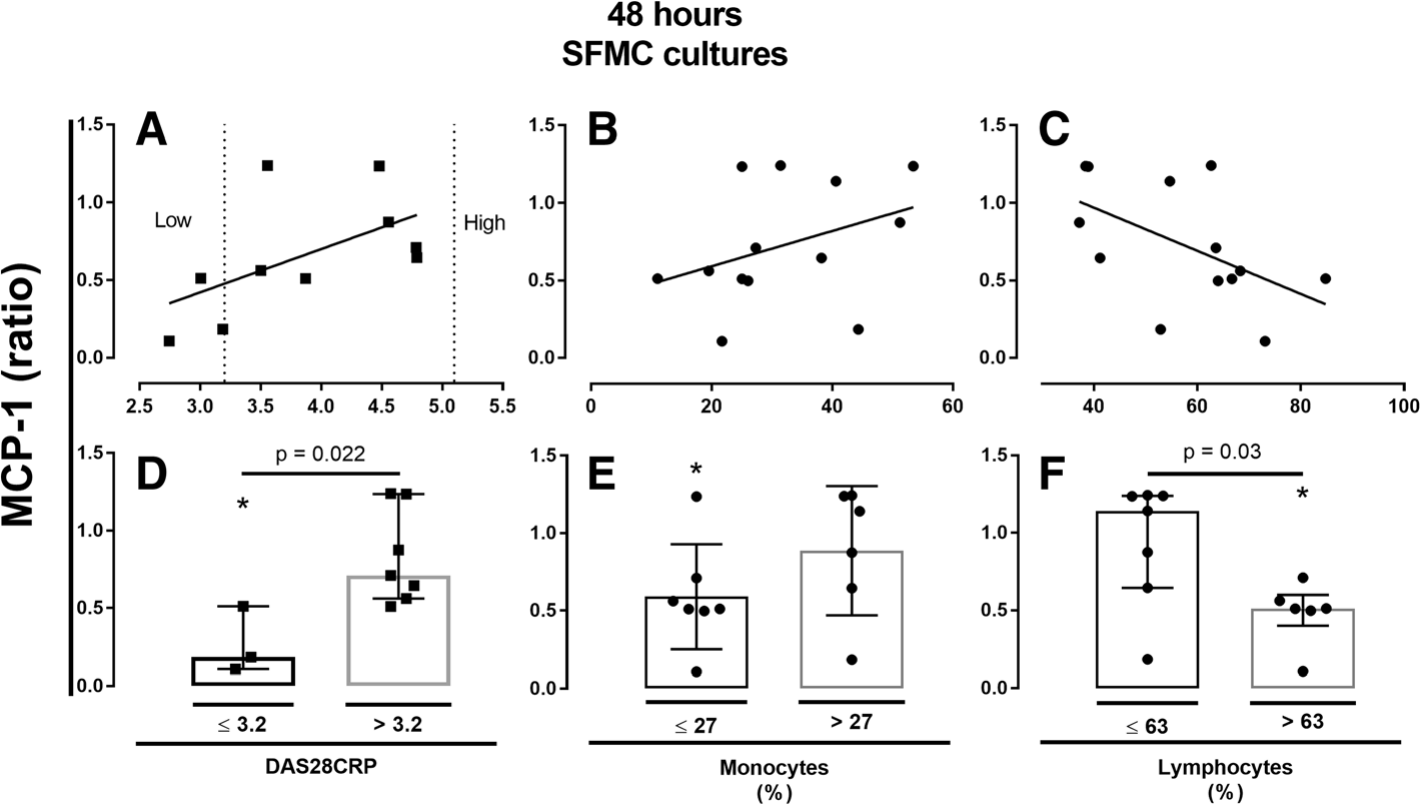
Ratio of MCP-1 in resveratrol (RSV) treated cultures (*n* = 11) plotted against DAS28CRP (**a**), and the percentage of monocytes (**b**) and lymphocytes (**c**) in synovial fluid of the donor. **d**-**f** depicts group comparisons as median and interquartiles. *p < 0.05 vs. negative control. MCP-1 was measured by ELISA. RSV concentration was 25 μM

The effect of RSV on MCP-1 concentration in 48 h SFMC cultures was assessed in the subgroup analyses as described in the methods section. In short, subgroup changes in MCP-1 concentrations were compared based on DAS28CRP and the lymphocyte/monocyte count in the SFMC cultures (Fig. 4).

In the subgroup analysis, RSV induced a significant MCP-1 decrease only in cultures from patients with DAS28CRP ≤ 3.2, *p* = 0.028, low monocytes count (≤ 27%, *p* = 0.019), and high lymphocyte count (> 63%, *p* = 0.002).

The median MCP-1 ratio was significantly decreased in patients with DAS28CRP ≤ 3.2 vs. > 3.2 (*p* = 0.022) and when lymphocyte count was > 63% compared with patients with ≤ 63% (*p* = 0.03).

In 48 h SFMC cultures similar visual tendency was present for MMP3 ratios but no statistical significant correlation. Thus, the effect of RSV was most potent in cultures from patients with low DAS28CRP and a high percentage of lymphocytes in the synovial fluid. However, the limited number of MMP3 results made subgroup analysis inappropriate.

The 21 days SFMC cultures showed no statistically significant correlation of MCP-1 or TRAP ratios, when plotted against DAS28CRP, monocyte count or lymphocyte count. Subgroup analysis was again inappropriate due to the small number of data.

### Resveratrol, methotrexate and adalimumab show similar MCP-1 lowering effects in 48 h SFMC cultures, but not 21 days SFMC cultures

**Fig. 5:**
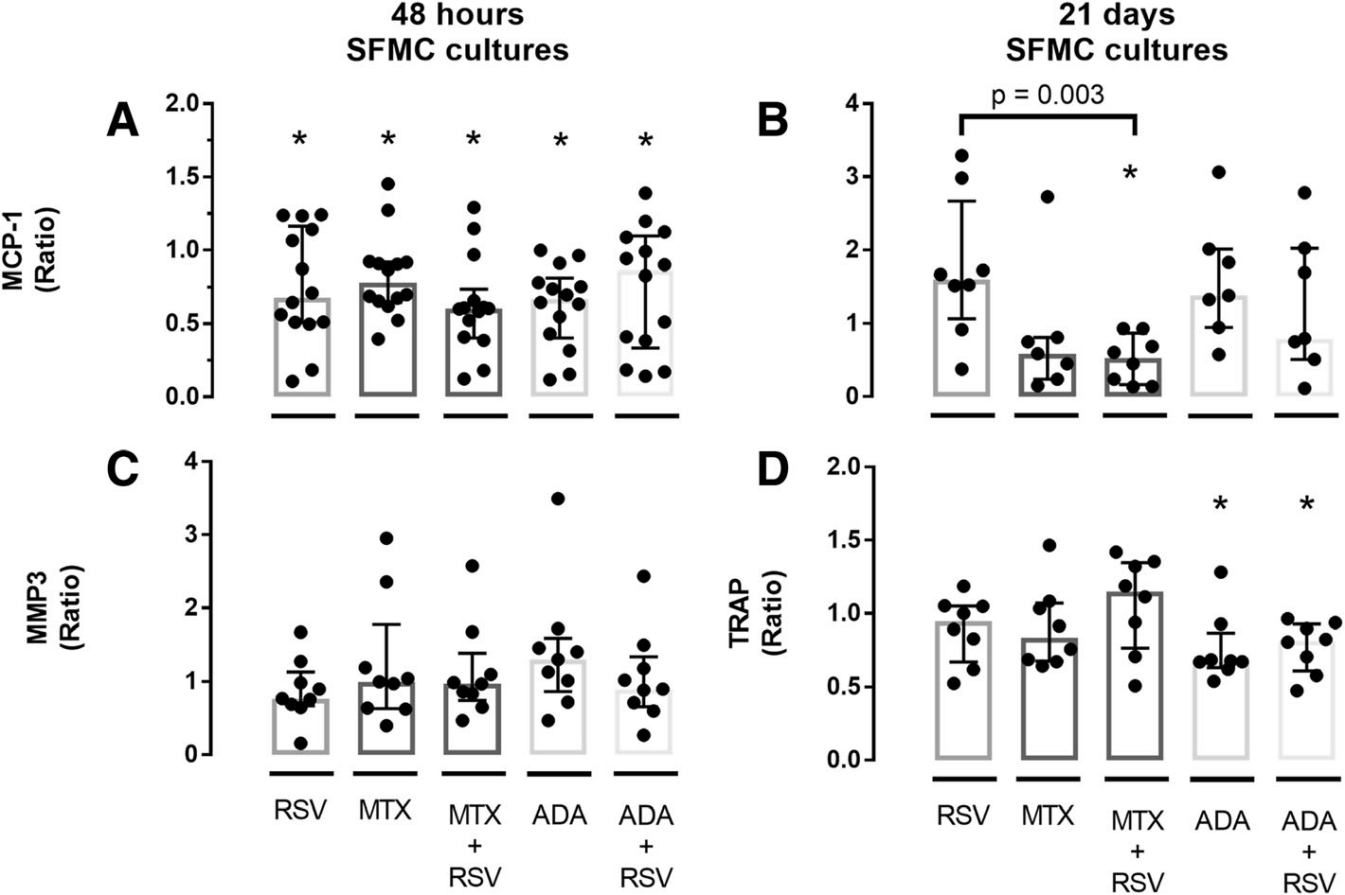
Ratios of MCP-1 (**a**, *n* = 14 and **b**, *n* = 8 (RSV and MTX + RSV) or *n* = 7 (MTX, ADA and ADA + RSV)) and MMP3 (**c**, *n* = 9) concentrations, and TRAP activity (**d**, *n* = 8) in supernatants from cultures with the noted substance or a combination of resveratrol (RSV), Ebetrex (MTX) and Adalimumab (ADA). Concentrations were: RSV; 25 μM, MTX; 0.5 μg/ml, Adalimumab; 5 μg/ml. MCP-1 and MMP3 were measured by ELISA and TRAP with an enzyme activity assay. Data is presented as a dot for each measurement; box and bars represent median and interquartile range. *p < 0.05 vs. negative control

To assess effects of different treatments, MCP-1 ratios (treated/untreated) from 48 h and 21 days SFMC culture were calculated. These ratios from RSV, MTX, MTX + RSV, adalimumab and adalimumab+RSV treatment are presented in Fig. 5. The ratios were then compared to identify differences in treatment effects.

In the 48 h SFMC cultures, MCP-1 ratios were significantly decreased by all the above-mentioned treatment conditions compared to no treatment (Fig. 5a). MMP3 ratios were not decreased significantly by any treatment (Fig. 5c), though RSV treatment resulted in the largest decrease (MMP3 ratio median = 0.77). MTX and adalimumab treatment did not result in a significantly different response compared to RSV in 48 h SFMC cultures and no additive effect of RSV was detected when including all cultures in the comparative analysis.

In 21 days SFMC cultures, MTX, MTX + RSV and adalimumab+RSV decreased median MCP-1 ratios, but only MTX + RSV produced a significant decrease. This was both when compared to the negative control (*p* = 0.008) and RSV alone (*p* = 0.0025). Adalimumab treatment produced a significant decrease in TRAP activity with and without RSV compared to no treatment (*p* = 0.023 and *p* = 0.008, respectively). No other treatment affected TRAP activity.

### Potential additive effects of RSV may be influenced by the level of disease activity and cellular subset in synovial fluid

**Fig. 6:**
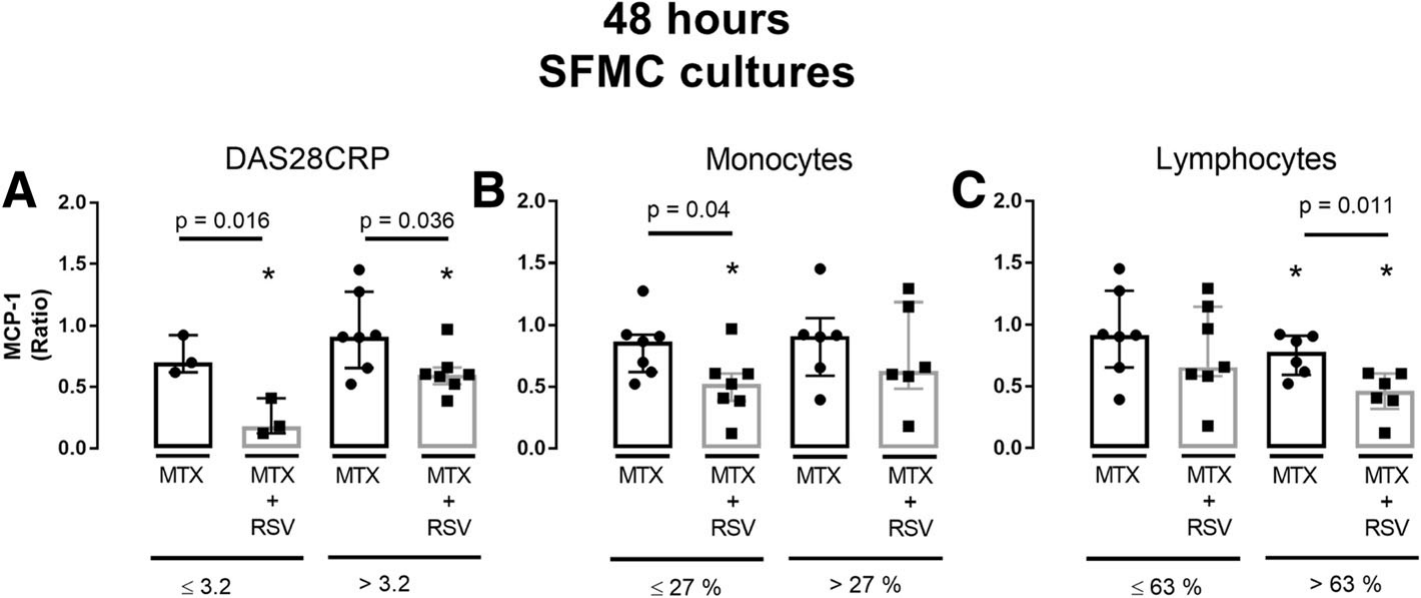
MCP-1 ratios as median and interquartiles in cultures treated with ebetrex (MTX) alone or in combination with resveratrol (RSV). Group comparisons were based on **a**) Disease Activity Score 28 (DAS28CRP), **b**) the percentage of monocytes and **c**) the percentage of lymphocytes in synovial fluid of the donor. *p < 0.05 vs. negative control. MCP-1 was measured by ELISA. Concentrations were: RSV = 25 μM and MTX = 0.5 μg/ml

In subgroup analysis of the 48 h SFMC cultures, MTX significantly decreased MCP-1 ratios in patients with high lymphocyte count (> 63%). RSV + MTX significantly lowered MCP-1 in patients with DAS28CRP ≤ 3.2, DAS28CRP > 3.2, low monocyte count (≤ 27%) and high lymphocyte count (> 63%); Fig. 6a-c.

Moreover, the addition of RSV to MTX treatment produced a significant lower MCP-1 ratio than MTX alone in the subgroup with DAS28CRP ≤ 3.2, *p* = 0.016, DASCRP > 3.2, *p* = 0.036, low monocyte count (≤ 27%, *p* = 0.04) and in cultures with high lymphocyte count (> 63%, *p* = 0.011).

**Fig. 7:**
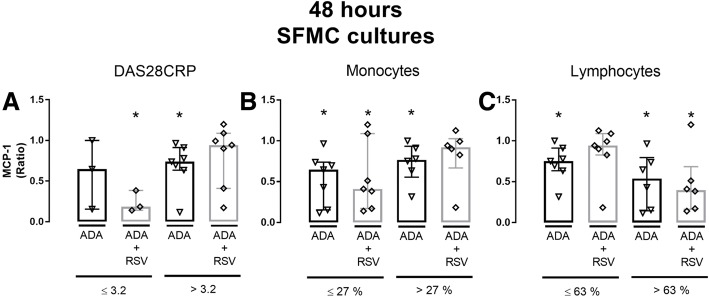
MCP-1 ratios as median and interquartiles in cultures treated with adalimumab (ADA) alone or in combination with resveratrol (RSV). Group comparisons were based on **a**) Disease Activity Score 28 (DAS28CRP), **b**) the percentage of monocytes and **c**) the percentage of lymphocytes in synovial fluid of the donor. *p < 0.05 vs. negative control. MCP-1 was measured by ELISA. Concentrations were: RSV = 25 μM and ADA = 5 μg/ml

Figure 7a-c presents subgroup MCP-1 ratio results (48 h SFMC cultures) for treatment with adalimumab alone or in combination with RSV. Both treatments produced significantly lower MCP-1 ratios in several subgroups (Fig. 7a-c) compared to no treatment. The greatest decrease in MCP-1 ratio was produced by adalimumab in combination with RSV in subgroups with DAS28CRP ≤ 3,2, low monocyte count (≤ 27%) and high lymphocyte count (> 63%).

None of these ratios were, however, significantly different compared with adalimumab treatment alone.

Subgroup analysis was not carried out for MMP3 ratios in 48 h SFMC cultures and for MCP-1 and TRAP ratios from 21 days SMFC cultures due to the smaller number of cell cultures.

## Discussion

A combination of two in vitro arthritis models was used to investigate the effects of RSV. RSV inhibited MCP-1 production in the 48 h inflammation model to a similar extent as MTX and adalimumab. This well-known anti-inflammatory property of RSV in vitro is in accord with previous reports [29, 34, 35]. Our results are to the author’s knowledge, however, the first to suggest that RSV’s anti-inflammatory effect may be influenced by the level of disease activity in patients with arthritis and composition of immune cells present in the cell cultures. Thus, RSV seemed to exert the greatest relative inhibition of MCP-1 in cultures from patients with low disease activity i.e. a DAS28CRP ≤ 3.2 or a synovial fluid infiltration dominated by lymphocytes.

RSV also resulted in a decrease of MMP3 ratio medians in 48 h SFMC cultures. However, this change was not significant as otherwise reported previously in fibroblast-like synoviocyte cultures from RA patients [8, 9]. Several parameters may offer an explanation to this. 1) The cellular composition of our models, i.e. SFMC may differ significantly from a fibroblast-like synoviocyte mono culture. 2) The mentioned studies only showed significant inhibition when cultures were additionally stimulated with TNF-α [8] or IL-1β [9], whereas our cultures received no additional stimulation in vitro. 3) The concentration of RSV may influence the effective inhibition of MMP3; Glehr et al. [9] used 100 μM versus our 25 μM, and Tian et al. [8] reported significant inhibition at 12.5, 25 and 50 μM. Despite differences, the two previous studies together with our data suggest an inhibitory effect of RSV on MMP3 production, and thereby a possible cartilage protective effect in relation to inflammatory arthritis.

RSV had no measurable effect on TRAP activity. This result is somewhat in conflict with previous reports were RSV is shown to diminish TRAP production in canine osteoclasts [36], and inhibit canine and murine osteoclastogenesis through various mechanisms [36, 37]. In contrast, our in vitro model received no additional stimulation, which may have limited our models’ ability to detect subtle changes in TRAP activity.

Osteoclasts are, however, not the only cell line of interest when evaluating RSV effects on arthritis. RSV has also been shown to attenuate pro-inflammatory processes in chondrocytes [30] and stimulates osteoblast differentiation [38]. Results that may give explanation for chondro-protective effects seen in osteoarthritic rabbits treated with intraarticular injections of RSV [39, 40].

Subgroup analysis of 48 h SFMC cultures showed RSV anti-inflammatory effect was most pronounced in cultures from patients with low disease activity and in patients with a synovial fluid dominated by lymphocytes. Our study was not designed to explain these differences. We therefore suggest further studies that investigate whether greater levels of systemic inflammation induce a different array of inflammatory pathways in these SFMCs, and whether pathways are affected differently by RSV at different levels of disease activity/inflammatory burden. It would also be interesting to see detailed investigations of the cellular subset, i.e. whether different lymphocyte subtypes are affected differently by RSV.

RSV has, to the author’s knowledge not been tested against or together with MTX or adalimumab in vitro or in vivo before. It is of great interest that the addition of RSV to MTX treatment showed significant additive effect especially in subgroups with low disease activity, or when the synovial fluid was dominated by lymphocytes. The effect in patients with DAS28CRP ≤ 3.2, is interesting because patients with low disease activity can still experience pain and discomfort. This encourages in vivo studies of the addition of RSV to MTX in patients with low disease activity. It should be mentioned that we did not have information about DAS28CRP in 4 patients which explains the differences in Fig. 5 (*n* = 14) and Fig. 6 (*n* = 10).

In addition to this, a previous study has reported that RSV protects against MTX induced oxidative stress in the small intestines of rats [41]. An interesting protective effect, since gastro-intestinal side effects from MTX are reported as a major reason for treatment discontinuation in RA patients [42]. Results from adalimumab treatment in the 48 h SFMC model, showed no significant additive effect of RSV, but did show same tendencies as with MTX.

Despite RSVs lipophilic nature and quick absorption from the small intestines, only relatively low amounts are found in the bloodstream after oral ingestion [43]. Studies explain this by a high rate of hepatic metabolism, which conjugates RSV with sulfates and glucuronides [1, 43]. Thus, a big challenge when discussing RSV as a possible drug candidate is drug delivery. Possible solutions could be a specific inhibition of the hepatic RSV metabolism or a delivery matrix that allows passage through the liver without major metabolism. Another possible delivery method is intraarticular injection of RSV. Animal studies have so far shown that intraarticular injected RSV reduce synovial hyperplasia and inflammation in an induced arthritis model [44], and attenuate if not prevent osteoarthritic progress in mice [45] and rabbits [39, 40].

As with all in vitro studies, the models used here have the fundamental limitation of only being a proxy to the in vivo situation, it seeks to investigate. This issue was addressed here by abstaining from additional stimulation of cultures, which in return may have diminished the study’s ability to detect subtle effects of RSV. The fact that this study in general have been limited to show trending effects of RSV and only few significant changes, may also be influenced by the relative small study population and unfortunate missing data from some cultures.

This study included a very heterogenous patient population with both RA and SpA patients on different disease modifying anti rheumatic drugs. RA and SpA both affect the synovial joints but also show very different clinical and immunological features. RSV treatment effect was not different when comparing SpA and RA in this study. Treatment with anti-TNFα or other disease modifying anti rheumatic drugs may affect immune cells even after isolation and thereby skew results from cultures. The potential influence of different treatments could not be studied due to the low sample size.

The study does, however, also possess strengths in its design and execution. First and foremost, the study combines both synovial fluid cells and osteoclasts involved in the osteoimmunological processes of inflammatory arthritis. The combination of models together with the different treatment conditions could allow interesting arguments across the pathological spectrum of inflammatory arthritis. The design furthermore permits the discovery of possible interactions between RSV, MTX and adalimumab, and enables it to suggest questions for future studies regarding RSVs effect at different levels of disease activity, inflammatory burden and on various cellular subsets.

## Conclusion

To conclude, this study confirms RSV as an anti-inflammatory agent in our ex vivo model of immune mediated inflammatory arthritis. An effect which our data suggest can work synergistic with MTX; especially in cases of low disease activity making RSV a possible supplemental treatment. As a result, we suggest future investigations into interactions and potential additive effects of RSV in MTX treatment of patients with immune mediated inflammatory arthritis.

## Additional file

Additional file 1: 48 h SFMC cultures – 21 days SFMC cultures. Photographic images showing similar cell density in cultures that were untreated or treated with resveratrol, methotrexate or adalimumab, and showing formation of multinucleated cells in 21 days SFMC cultures. (PDF 443 kb)

## Abbreviations

DAS28CRP: Disease activity score 28
ELISA: Enzyme-linked immunosorbent assay
IL-1β: Interleukin 1 beta
MCP-1: Monocyte chemoattractant protein 1
MMP3: Matrix metalloproteinase 3
MTX: Methotrexate
RA: Rheumatoid arthritis
RSV: Resveratrol
SFMC: Synovial fluid mononuclear cells
SpA: Spondyloarthritis
TNF-α: Tumor necrosis factor alfa
TRAP: Tartrate resistant acid phosphatase
