# Supplementary material for: Resveratrol displays anti-inflammatory properties in an ex vivo model of immune mediated inflammatory arthritis
Source: BMC Rheumatol. 2018 Oct 10;2:27. doi: 10.1186/s41927-018-0036-5 (PMC6390607; doi:10.1186/s41927-018-0036-5)
Supplement: Supplementary file 1 — 48 h SFMC cultures – 21 days SFMC cultures. Photographic images showing similar cell density in cultures that were untreated or treated with resveratrol, methotrexate or adalimumab, and showing formation of multinucleated cells in 21 days SFMC cultures. (PDF 443 kb) [file 41927_2018_36_MOESM1_ESM.pdf]

48 hours

SFMC cultures

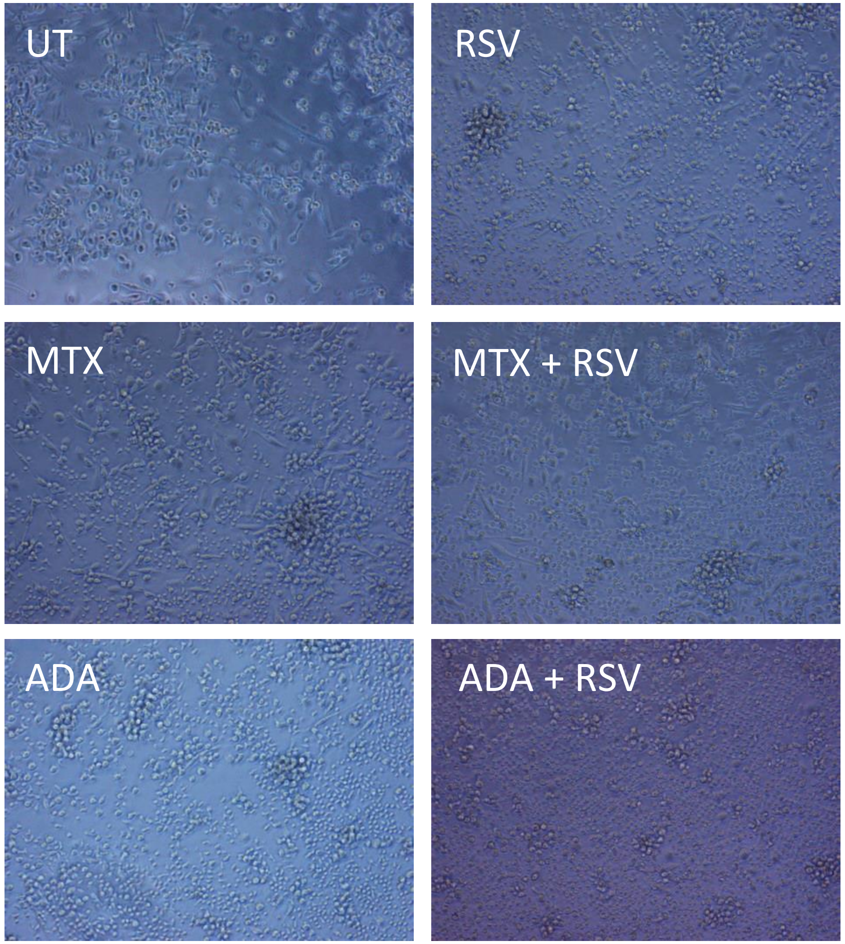

21 days

SFMC cultures

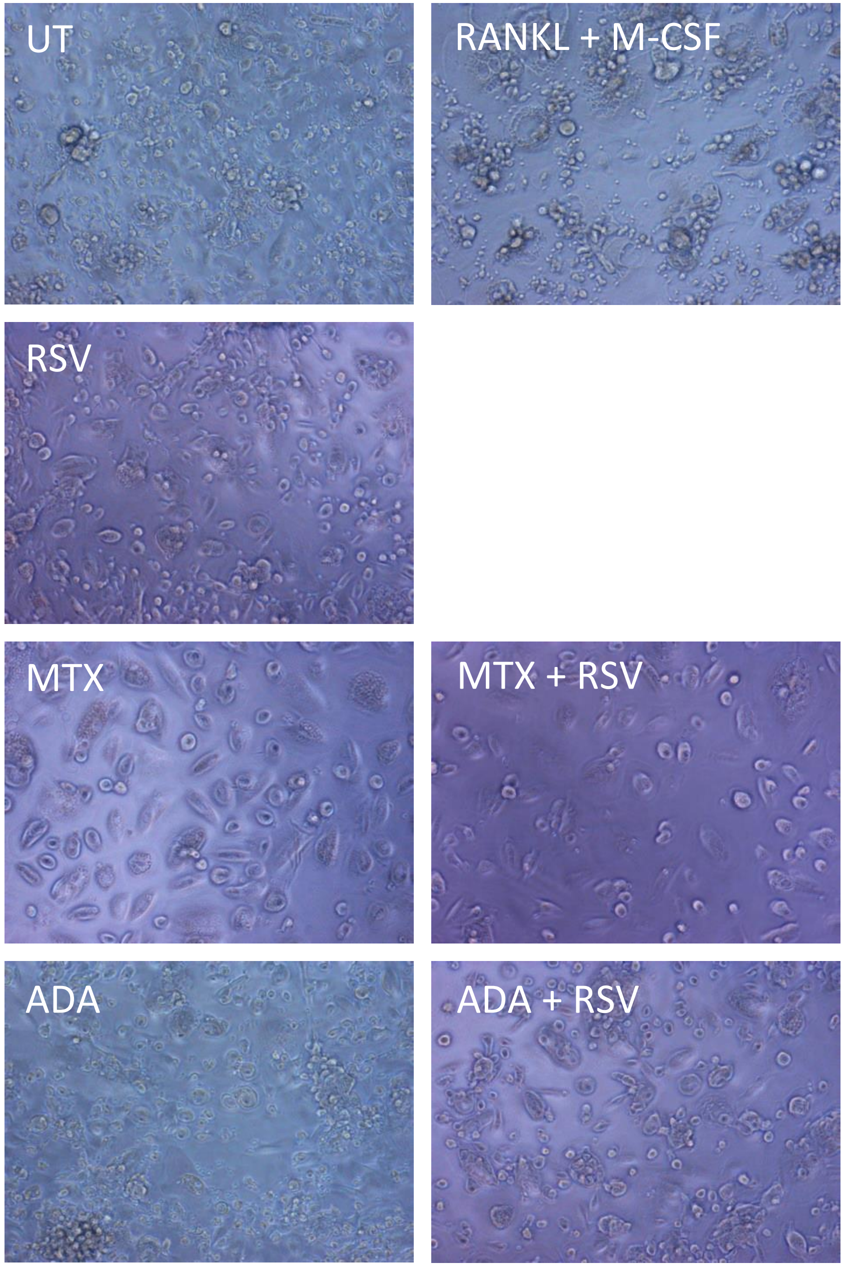

Additional file 1 Microscope images (x10) of 48 hours and 21 days SFMC after incubation period. No immediate cell loss was detected after treatment. Multinucleated cells were present in 21 days SFMC cultures. UT: untreated (negative control), RANKL + M- SCF: positive control, RSV: resveratrol 25 µM, MTX: Ebetrex 5 µg/ml, ADA: Adalimumab 5 µg/ml.
